# Extract from *Astragalus membranaceus* inhibit breast cancer cells proliferation via PI3K/AKT/mTOR signaling pathway

**DOI:** 10.1186/s12906-018-2148-2

**Published:** 2018-03-09

**Authors:** Ruijuan Zhou, Hongjiu Chen, Junpeng Chen, Xuemei Chen, Yu Wen, Leqin Xu

**Affiliations:** 1 Department of Chest and Breast Surgery, Xiamen Hospital of Traditional Chinese Medicine, Fujian University of Traditional Chinese Medicine, 1739 Xianyue Road, Xiamen, 361009 People’s Republic of China; 2 Department of Pharmacy, Xiamen Hospital of Traditional Chinese Medicine, Fujian University of Traditional Chinese Medicine, 1739 Xianyue Road, Xiamen, 361009 People’s Republic of China; 3 Department of Science and Education, Xiamen Hospital of Traditional Chinese Medicine, Fujian University of Traditional Chinese Medicine, 1739 Xianyue Road, Xiamen, 361009 People’s Republic of China

**Keywords:** *Astragalus membranaceus*, Extract, Breast cancer, Apoptosis, PI3K, AKT

## Abstract

**Background:**

*Astragalus membranaceus* (AM) is a commonly used herb in traditional Chinese medicine (TCM), which has been used as an essential tonic to treat various diseases for more than 2000 years. In this study, we aimed to investigate the biological effects of extract from AM on breast cancer cell and its mechanism.

**Methods:**

To prepare the extract, dried AM were ground and extracted with water extraction-ethanol supernatant method. Then the main isoflavones in the extract was detect by HPLC analysis. Furthermore, the anti-proliferative activity of AM extract was examined by MTT assay and morphological observation. Cell apoptosis was evaluated with flow cytometric analysis. The expressions of total and phosphorylated PI3K, GS3Kβ, Akt and mTOR were determined by western blot analysis.

**Results:**

HPLC analysis demonstrated that AM extract contained with four kinds of isoflavones, campanulin, ononin, calycosin and formononetin. The MTT test and morphological observation indicated that cells proliferation of MCF-7, SK-BR-3 and MDA-MB-231were inhibited by AM extract in a dose dependent manner. Furthermore, flow cytometric analysis displayed that after treated with 25 μg/ml and 50 μg/ml AM extract, apoptosis of breast cancer cells was significantly increased as compared with DMSO and blank control group (all *p* < 0.05). Western blot analysis found that the level of p-PI3K, p-GS3Kβ, p-Akt, and p-mTOR were significantly decreased, but the level of total-mTOR was observably increased as compared with DMSO control group.

**Conclusions:**

Taken together, the inhibited cell proliferation and induced cell apoptosis effect of AM extract via PI3K/AKT/mTOR pathway confirmed the anti-tumor potential of AM. Therefore, our findings provide a new insight into anti-cancer effect of AM extract as a promising agent in breast cancer treatment.

**Electronic supplementary material:**

The online version of this article (10.1186/s12906-018-2148-2) contains supplementary material, which is available to authorized users.

## Background

Breast cancer is the most threatening health problem for women worldwide in incidence and mortality. In 2012, an estimated more than 1.6 million new cases were diagnosed with breast cancer globally with 521,907 women died due to breast cancer [1]. Surgical resection is the most effective treatment for breast cancer. Chemotherapy, radiotherapy and endocrine therapy are usually used to eliminate remaining tumor cells, inhibit tumor growth, and reduce breast cancer recurrence. These therapies improve long-term survival and quality of life for breast cancer patient [2]. Unfortunately, some patients experience treatment-related adverse effects or become resistant to these reagents. Therefore, finding bioactive natural products may provide an alternative strategy in breast cancer treatment.

Aberrant activation of the PI3K/Akt/mTOR signaling pathway has been shown in numerous cancers, including breast cancers [3]. Accumulating evidence indicates that the PI3K/AKT/mTOR signaling pathway plays a pivotal role in the regulation of breast cancer growth, survival, and motility as well as the acquisition of drug resistance, and there are now extensive data indicating that various components of this pathway as potential molecular targets for breast cancer treatment [4]. Therefore, PI3K/AKT/mTOR signaling pathway is considered as an attractive target for the development of new anticancer agents that could be used alone or in combination with other targeted therapies for treating breast cancer patients.

Over the past few decades, researchers have studied many biological properties of a number of promising plants and herbs. *Astragalus membranaceus* (Radix Astragali or “Huang Qi”, AM) has been used for medicinal purposes in traditional Chinese medicine over 2000 years. It is well-known for its vital-energy tonifying, skin reinforcing, diuretic, abscess-draining and tissue generative actions [5]. In practice, AM is often combined with other herbs, such as angelica, poria and ginseng, in various complex prescription formulas [6–8]. In our previous studies, we found that Yiqi formula (AM combined with poria) enhanced the antitumor effects of erlotinib on triple-negative breast cancer xenografts [9]. Although AM is usually combined with other herbs, it can be taken separately by itself. It contains several types of bioactive compounds including ploysaccharides, flavonoids, and saponins [10–15]. In recent years, AM has been investigated in treating various cancers [16–22]. However, the effects of AM extract on breast cancer are still unknown and very limited information is available on the mechanism responsible for the anticancer effects.

The purpose of this present work was to extract AM through the water extraction-ethanol supernatant method and determine its anti-proliferative effect on three distinct breast cancer cell lines, MCF-7 (ER+), SK-BR-3 (HER2+) and MDA-MB-231 (triple-negative). Our results demonstrated that AM extract showed a cytotoxic effect on breast cancer cells, and its mechanism of anticancer action seemed to induce breast cancer cell apoptosis via PI3K/AKT/mTOR signaling pathway.

## Methods

### Chemicals, reagents, and antibodies

Campanulin, ononin, calycosin and formononetin were purchased from Phytomarker Ltd. (Tianjin, China). Penicillin, streptomycin, phosphate-buffered saline (PBS), trypsin-EDTA, DMEM, RPMI 1640 and fetal bovine serum (FBS) were obtained from Invitrogen (Carlsbad, CA, USA). 3-(4,5-dimethylthiazol-2-yl)-2,5-diphenyltetrazolium bromide (MTT) and Annexin V/propidium iodide (PI) were purchased from Sigma (St. Louis, MO, USA). Fluorescein isothiocyanate (FITC)-labeled secondary antibody was obtained from Invitrogen (Carlsbad, CA, USA). Antibodies against PI3K, p-PI3K, GS3Kβ, p-GS3Kβ, AKT, p-AKT, mTOR, p-mTOR and β-actin were purchased from Cell Signaling Technology (Danvers, MA, USA). Goat anti-rabbit and goat anti-mouse peroxidase conjugated secondary antibodies were purchased from Bio-Rad (Hercules, CA, USA).

### Plant material and extract preparation

**Fig. 1:**
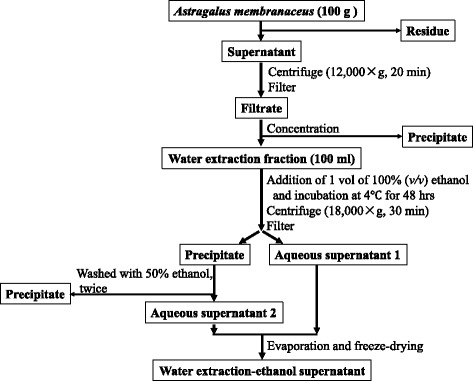
Schematic depiction of extraction method from AM

Root of AM in dried form of preeminent grade was bought from Luyan pharma Co. Ltd. (Fuzhou, China). The scheme of the extraction procedure was shown in Fig. 1. Briefly, for the water extraction, AM were extracted with 100 °C distilled water for 4 h at a ratio of 1:10 (*w*/*v*). This procedure was repeated twice. The aqueous extract was centrifuged at 12000×g for 20 min and filtered through a filter paper (GF/A, 47 mm; Whatman, UK). Then the water extraction was added to 1 volumes of 100% ethanol and then stored at 4 °C for 48 h. The precipitate and aqueous supernatant 1 were separated and collected by centrifugation at 18,000×g for 30 min. Then the precipitate was washed with 50% ethanol for twice and filtered to obtain the aqueous supernatant 2. The aqueous supernatant 1 and supernatant 2 were added together, then concentrated in a vacuum evaporator and lyophilized to obtain the water extraction-ethanol supernatant.

### High pressure liquid chromatography (HPLC) analysis

Analysis of isoflavones in AM extract were performed using a liquid chromatograph (Series 1100, Agilent Technologies, Palo Alto, CA, USA), consisting of ultraviolet (UV) detector, a dual pump, an autosampler, an ELSD (Alltech Associates, Deer field, IL, USA), a ZORBAX ODS C_18_ column and a guard column using HP Chem Station software (Agilent Technologies). The column temperature was maintained at a constant 40 °C, and the mobile phase flow rate was 1 ml/min. The mobile phase consisted of acetonitrile and H_2_O, which were applied in a gradient of acetonitrile as follows: 0–10 min, 25–35%; 10–25 min, 35–45%; 25–35 min, 45–55%; 35–42 min, 55–71%. UV detection was performed at 251 nm, and sample size was 20 μl.

### Cell culture and maintenance

MCF-7, SK-BR-3 and MDA-MB-231 (human breast cancer) cells were procured from American Type Culture Collection (ATCC, Manassas, VA, USA) and maintained under the conditions as previously described [9, 23]. MCF-7 cell line (ER+, HER2−) was cultured in Dulbecco’s modified Eagle medium (DMEM, Hyclone, UT, USA) supplemented with 10% fetal bovine serum (FBS, Hyclone) and 1% penicillin/streptomycin (Invitrogen, NY, USA). SK-BR-3 cell line (ER−, PR−, HER2+) was maintained in McCoy’s 5a medium supplemented with 10% FBS and 1% penicillin/streptomycin. MDA-MB-231 cell line (a triple-negative breast cancer (TNBC) cell line, ER−, PR−, and HER2−) was maintained in DMEM containing 10% FBS with 1% penicillin/streptomycin. All cells were grown as monolayers and were maintained in a humidified CO_2_ incubator at 37 °C in 5.0% CO_2_ and 95.0% air. The media was changed every two or three days. Cells were detached with 0.25% Trypsin-EDTA in PBS.

### Cell proliferation assay

To assess the effects of AM extract on breast cancer cells growth, MTT assay was performed as previously described [23]. MCF-7, SK-BR-3 and MDA-MB-231 cells were seeded into 96-well plates and cultured at a density of 5 × 10^3^ cells per well. After 24 h of incubation, the cells were treated with vehicle (0.1% DMSO) or different concentrations of AM extract (100, 50, 25 μg/ml) for 48 h. Then MTT solution was added to each well (1.2 mg/ml) and incubated at 37 °C for 4 h. The concentration of MTT-formazan product dissolved in DMSO was estimated by measuring absorbance at 490 nm in an absorbance micro-plate reader.

### Morphological observation under inverted microscope

In order to investigate the effect of AM extract on cell morphology, breast cells were seeded in 24-well plates (10^5^ cells/well). After 24 h of incubation, the cells were treated with different concentrations of AM extract for 48 h. Then breast cancer cells were examined under inverted microscope (OLYMPUS IX70-S8F, Olympus Optical Co., Ltd., Japan) and photographs were taken.

### Detection of apoptosis via FITC-Annexin V/PI staining

For Annexin V/propidium iodide (PI) assay, MCF-7, SK-BR-3 and MDA-MB-231 cells were stained with Annexin V-fluorescein isothiocyanate and PI and evaluated for apoptosis by flow cytometry according to the manufacturer’s protocol (BD PharMingen, San Diego, CA). In brief, the breast cancer cells were seeded into six-well plates at a density of 2 × 10^5^cells/well. After 24 h incubation, breast cancer cells were exposed to 50 μg/ml of AM extract for 24 h. Afterward, breast cancer cells were harvested with 0.25% Trypsin–EDTA and washed twice with phosphate-buffered saline (PBS), and then stained with 5 μl of Annexin V-fluorescein isothiocyanate and 10 μl of PI (5 μg/ml) in 1× binding buffer (10 mM HEPES, pH 7.4, 140 mM NaOH, and 2.5 mM CaCl_2_) for 15 min at room temperature in the dark. Labeled cells were determined using a FACScan Cytometer (BD Biosciences, San Jose, CA). Over 10,000 cells of each sample were counted and there after the percentage of apoptotic cell death was quantitatively analyzed. Three independent experiments were performed.

### Western blot analysis

Western blot analysis was performed as previously described by our group [9, 23, 24]. After treatment with various dosages of AM extract for 48 h, the breast cancer cells (MCF-7, SK-BR-3 and MDA-MB-231) were lysed for 15 min with RIPA buffer containing protease and phosphatase inhibitors. The protein concentrations were measured with a BCA kit (Beyotime, China). Equal amounts of protein were separated by sodium dodecyl sulfate-polyacrylamide gel electrophoresis (SDS-PAGE) and transferred to a polyvinylidene fluoride (PVDF) membrane. The membrane was blocked with a solution containing 5% nonfat dry milk TBST buffer (20 mM Tris–HCl, pH 7.4, 150 mM NaCl and 0.1% Tween 20) for 1 h. The indicated primary antibodies were incubated overnight at 4 °C, washed, and monitored by immunoblotting using a DyLight 800-conjugated secondary antibody. The membrane was scanned using a LI-COR Infrared Imaged Odyssey (Gene Company Limited).

### Statistical analysis

All data were presented as the means ± standard deviation (S.D.) of three independent experiments. Statistical analysis was performed by Student’s t-test or one-way analysis of variance (ANOVA). In all cases, *p* < 0.05 was considered statistically significant.

## Results

### Identification of crude isoflavones in AM extract with HPLC analysis

**Fig. 2:**
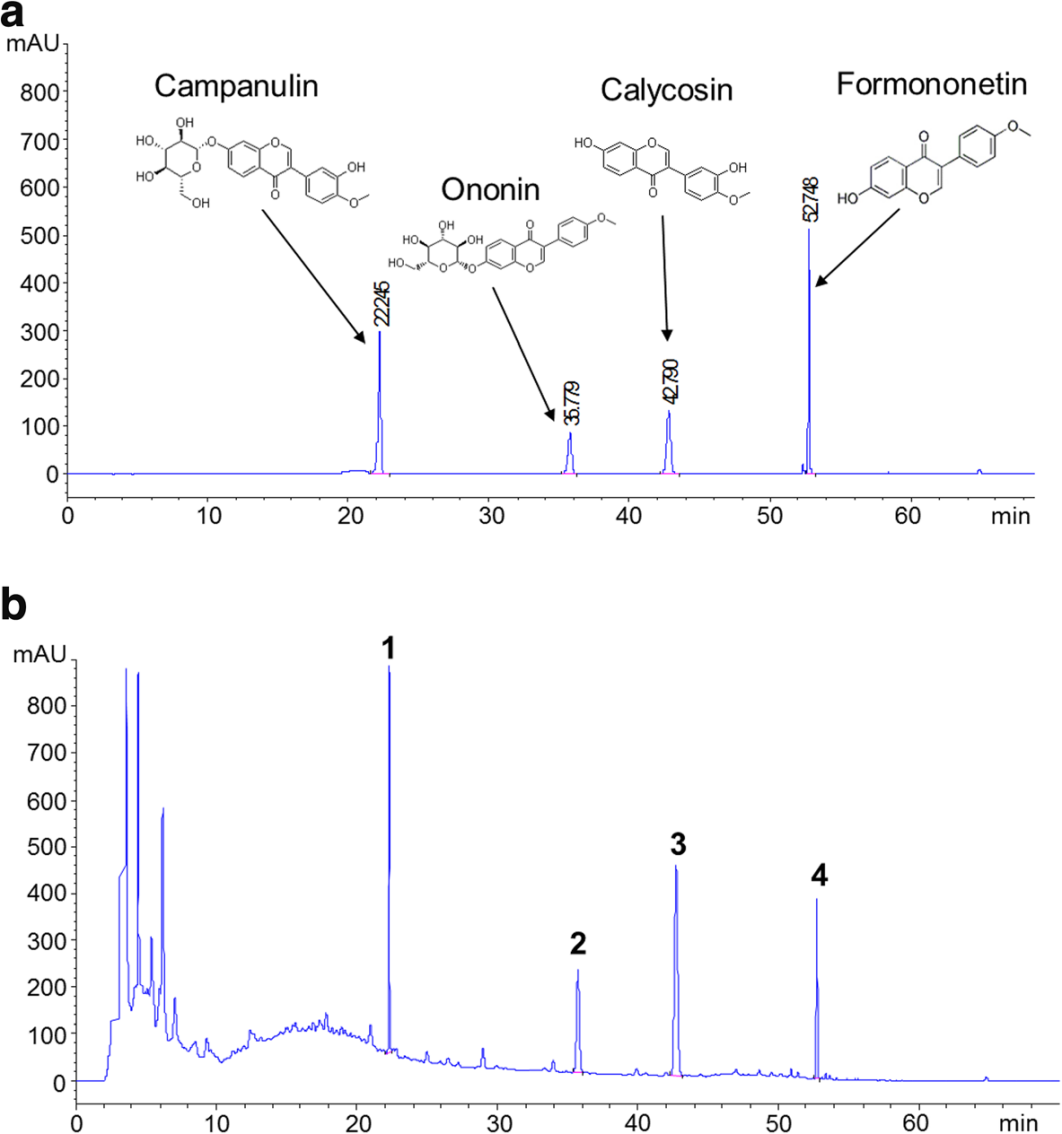
HPLC profile of AM extract. **a** HPLC chromatogram of mixed standard solutions, campanulin, ononin, calycosin, and formononetin. **b** HPLC chromatogram of AM extract. Campanulin, ononin, calycosin, and formononetin were detected in AM extract. The retention times were 22.245 min (peak 1), 36.779 min (peak 2), 42.79 min (peak 3), and 52.748 min (peak 4), respectively

For detecting the isoflavones in AM extract, HPLC analysis was used to compare AM extract with standard components, campanulin, ononin, calycosin, and formononetin (Fig. 2a). Campanulin, ononin, calycosin, and formononetin were detected in AM extract by HPLC analysis at wavelengths of 251 nm. The retention times were 22.245 min, 36.779 min, 42.79 min, and 52.748 min, respectively (Fig. 2b).

### AM extract suppressed proliferation of breast cancer cell

**Fig. 3:**
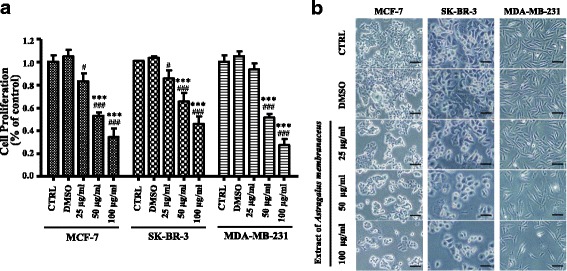
Cytotoxic effects of AM extract on three distinct breast cancer cell lines. **a** After treated with different concentration of AM extract for 48 h, the cell proliferation was evaluated with MTT assay. Values are the mean ± S.D. of triplicate determinations of three independent experiments. ***p < 0.001, compared with blank control group (CTRL); ^#^*p* < 0.05, ^###^*p* < 0.001, compared with DMSO group. **b** Morphological effect of AM extract on breast cancer cells

The cytotoxic effect of AM extract was evaluated by MTT assay. As indicated in Fig. 3a, AM extract markedly inhibited breast cancer cell growth in a dose dependent manner in 48 h. These results were further confirmed by morphological examination (Fig. 3b).

### AM extract induced apoptosis in breast cancer cells

**Fig. 4:**
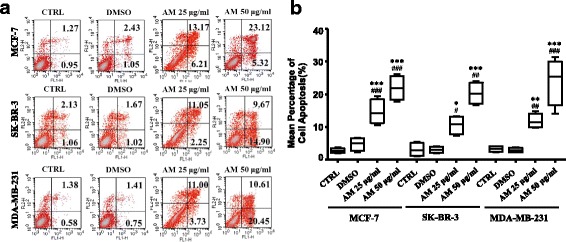
Analysis of cell apoptosis induced by AM extract in three distinct breast cancer cell lines. Breast cancer cells were treated with 50 μg/ml of AM extract for 24 h, and cell apoptosis was assessed by flow cytometry with FITC-Annexin V/PI Staining. The data are representative of three independent experiments carried out under the same conditions. *p < 0.05, **p < 0.01, ***p < 0.001, compared with blank control group (CTRL); ^#^*p* < 0.05, ^##^*p* < 0.01, ^###^*p* < 0.001, compared with DMSO group

Annexin V/PI staining was used to determine whether the action of AM extract was associated with apoptosis or not. As shown in Fig. 4, AM extract was strongly effective on three cell types. Breast cancer cells treated with 25 μg/ml and 50 μg/ml of AM extract for 24 h showed a marked increase in the number of cells apoptosis (Fig. 4).

### Effects of AM extract on the PI3K/AKT signaling pathway

**Fig. 5:**
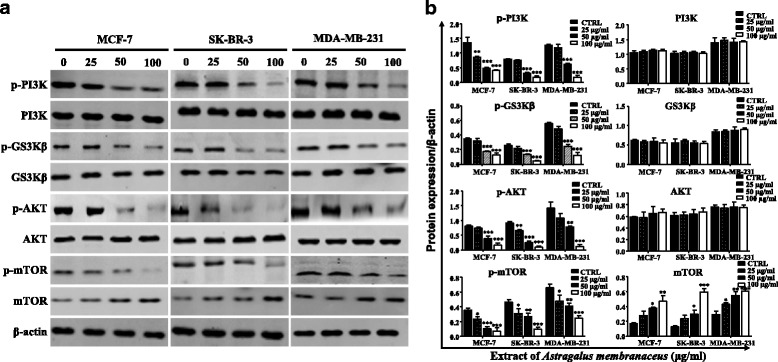
Protein expressions of PI3K, GS3Kβ, Akt, mTOR, p-PI3K, p-GS3Kβ, p-Akt, and p-mTOR in breast cancer cells by western blot analysis. β-actin was used as a protein loading control. *p < 0.05, **p < 0.01, ***p < 0.001, compared with blank control group (CTRL)

To investigate the effect of AM extract on PI3K/AKT signaling pathway in breast cancer cells, we performed western blot analysis of phosphorylated and total- PI3K, GS3Kβ, Akt and mTOR. Our results showed that AM extract could inhibit the expressions of p-PI3K, p-GS3Kβ, p-AKT and p-mTOR in a dose-dependent manner (Fig. 5).

## Discussion

In the present study, we demonstrated that extract from AM, with water extraction-ethanol supernatant method, has anti-proliferative activity on breast cancer cell. This extract contented four kinds of isoflavones, campanulin, ononin, calycosin, and formononetin. As part of inhibitory effect on breast cancer cells proliferation, the AM extract decreased the expression of p-PI3K, p-GS3Kβ, p-AKT and p-mTOR, and led to breast cancer cell apoptosis.

Since many bioactive constituents in plants are flavonoids, polysaccharides and saponins [13]. Flavonoids are found in most parts of plants and have been shown to have multiple biological activities such as anti-cancer, anti-inflammation, antibacteria, antivirus, and immune-stimulation [25, 26]. A recent published review by Auyeung K. K. et al. presents that the AM contains flavonoids within the range of 0.5–3.0 mg/g. A total of 12 different flavonoids can be isolated from AM. These flavonoids include isoflavonones, isoflavans, pterocarpans, flavonones, and chalcones, of which isoflavones are the major constituents [5]. It has been reported that some of isoflavones from AM, such as calycosin and formononetin have inhibited cancer cell proliferation and metastasis effect [17, 23, 25, 27, 28]. The most common extraction method in herbal medicine is to boil the herb in hot water, which is called decoction. Decoction has long been used in traditional Chinese medicine, which is suitable for extracting heat-stable compounds, hard plants materials (e.g. roots and barks) and usually used for extracting oil-soluble compounds [29]. It has been reported that flavonoids were stable during heating reflux in water bath for 30 min and they were better extracted in water-alcoholic solution than by pure solvent [30]. So in this study, we used the water extraction-ethanol supernatant method to extract flavonoids from AM. Due to limited experimental conditions, we only detected four isoflavonoids, campanulin, ononin, calycosin, and formononetin in the AM extract (Fig. 2). Except these four compounds, the extract may content other compounds, such as polysaccharides and saponins. Because, there are many unknown chromatographic peaks in the AM extract (Fig. 2b). We will analyze the other compounds in this extract, detect their biology activities in further experiments and compare the content of active components with different extraction methods.

The anti-proliferative effect of AM extract on different breast cancer cell lines was present in Fig. 3. The presented results showed that exposure of breast cancer cells to AM extract for 48 h resulted in growth inhibition in a dose-dependent manner. Moreover, treatment with 50 μg/ml AM extract significantly promoted the apoptosis of breast cancer cells (Fig. 4). Thus, these results suggest that the AM extract inhibits the proliferation of breast cancer cells by the induction of apoptosis. Induction of apoptosis is thus considered as a strategy for cancer control. As published data showed that AM extract inhibited the growth of various cancer cells in vitro, such as colon cancer cells [12, 31], hepatocellular carcinoma [18, 21, 32], gastric cancer cell [16] and non-small lung cancer cell [33]. Furthermore, total flavonoids from AM have a significant inhibitory effect on human hepatocellular carcinoma BEL-7402 cell [34] and erythroleukemia K562 cell in vitro [25]. To date, there are two main apoptotic pathways: the extrinsic or death receptor pathway and the intrinsic or mitochondrial pathway [35]. It has been reported that, AM induced hepatocellular carcinoma cell [18] and gastric cancer cells [16] apoptosis via extrinsic or induced colon cancer cells via intrinsic pathway [31]. However in this study, we only observed the phenomena of AM extract on breast cancer cell proliferation and cell apoptosis. We will analyze the expression of death receptors, such as FasL/FasR, TNF-α/TNFR1, and Bcl-2 family such as Bcl-2, Bcl-x and cytochrome c in our next experiment, to confirm the effect of AM extract on inducing breast cancer cell apoptosis via which pathway.

To investigate the mechanisms by which AM extract inhibit cell growth and promoted apoptosis in breast cancer cells, we analyzed PI3K/Akt/mTOR signaling pathways after treatment with the AM extract. The PI3K/Akt/mTOR is a major intracellular signaling pathway, which plays a key role in cell proliferation, growth, migration, metabolism and apoptosis [36]. Aberrant activation of the PI3K/Akt/mTOR pathway is found in many types of cancer including breast cancer [37, 38]. The recent development and clinical testing of PI3K/Akt/mTOR inhibitors have led to the conclusion that targeting the PI3K/Akt/mTOR pathway is a promising approach for the treatment of breast cancer [39]. After treatment with the AM extract, the expression levels of p-PI3K, p-GS3Kβ, p-Akt and p-mTOR were effectively suppressed (Fig. 5). In light of the key role of PI3K/Akt/mTOR signaling pathway in governing apoptosis, our study showed that inhibition of PI3K/Akt/mTOR pathway by the AM extract increase the apoptosis of breast cancer cells. This results indicated that breast cancer cells apoptosis induced AM extract were related to the inhibition of PI3K signaling pathway. However, it is uncertain whether AM extract induces breast cancer cells apoptosis only through the PI3K/Akt/mTOR signaling pathway or by other pathways. We will use siRNA experiments or PI3K/Akt inhibitor in our further study.

## Conclusions

In conclusion, in this study, we demonstrate that the extract from AM with water extraction-ethanol supernatant method inhibit cell growth and induce apoptosis in cultured breast cancer cells. The effect of AM extract to suppress breast cancer cells growth was associated with its ability to inhibit PI3K/Akt/mTOR activity. These results suggest that the AM could provide an alternative strategy for breast cancer patients. Further studies are needed to identify all components in the AM extract and determine in vivo effects of this extract in animal models, in order to better evaluate the therapeutic potential of AM.

## Additional files

Additional file 1: **Figure S1.** Cytotoxic effects of AM extract on three breast cancer cell lines for 24 h. After treated with different concentration of AM extract for 24 h, the cell prolieration was evaluated with MTT assay. Values are the mean ± S.D. of triplicate determinations of three independent experiments. **p < 0.01, ***p < 0.001, compared with blank control group (CTRL); ^#^*p* < 0.05, ^##^*p* < 0.01, ^###^*p* < 0.001, compared with DMSO group. (PPTX 92 kb)

Additional file 2: **Figure S2.** Analysis of cell apoptosis induced by AM extract in MCF-7 for 48 h. MFC-7 breast cancer cells were treated with 25 μg/ml and 50 μg/ml of AM extract for 48 h, and cell apoptosis was assessed by flow cytometry with FITC-Annexin V/PI Staining. (PPTX 262 kb)

## Abbreviations

Akt: Protein kinase B
AM: Astragalus membranaceus
ATCC: American Type Culture Collection
DMEM: Dulbecco’s modified Eagle medium
ER: Estrogen receptor
FBS: Fetal bovine serum
GS3Kβ: Glycogen synthase kinase-3 beta
HER2: Human epidermal growth factor receptor 2
HPLC: High pressure liquid chromatography
mTOR: Mammalian target of rapamycin
MTT: 3-(4,5-dimethylthiazol-2-yl)-2,5-diphenyltetrazolium bromide
PBS: Phosphate-buffered saline
PI: Propidium iodide
PI3K: Phosphatidylinositol-4,5-bisphosphate 3-kinase
PR: Progesterone receptor
TNBC: Triple-negative breast cancer

## References

[1] Ghoncheh M, Pournamdar Z, Salehiniya H (2016). Incidence and mortality and epidemiology of breast cancer in the world. Asian Pacific journal of cancer prevention : APJCP.

[2] Chew HK (2001). Adjuvant therapy for breast cancer: who should get what?. West J Med.

[3] Cidado J, Park BH (2012). Targeting the PI3K/Akt/mTOR pathway for breast cancer therapy. J Mammary Gland Biol Neoplasia.

[4] Guerrero-Zotano A, Mayer IA, Arteaga CL (2016). PI3K/AKT/mTOR: role in breast cancer progression, drug resistance, and treatment. Cancer Metastasis Rev.

[5] Auyeung KK, Han QB, Ko JK (2016). Astragalus membranaceus: a review of its protection against inflammation and gastrointestinal cancers. Am J Chin Med.

[6] Woo SM, Choi YK, Cho SG, Park S, Ko SG: A new herbal formula, KSG-002, Suppresses Breast Cancer Growth and Metastasis by Targeting NF- kappa B-Dependent TNF alpha Production in Macrophages. Evidence-based complementary and alternative medicine : eCAM 2013, 2013:728258. 10.1155/2013/728258 PMC3683439 23818931

[7] Choi YK, Cho SG: Herbal extract SH003 suppresses tumor growth and metastasis of MDA-MB-231 breast cancer cells by inhibiting STAT3-IL-6 signaling. 2014, 2014:492173. 10.1155/2014/492173 PMC4058205 24976685

[8] Choi YY, Kim MH, Hong J, Kim K: Effect of Dangguibohyul-Tang, a Mixed Extract of Astragalus membranaceus and Angelica sinensis, on Allergic and Inflammatory Skin Reaction Compared with Single Extracts of Astragalus membranaceus or Angelica sinensis. 2016, 2016:5936354. 10.1155/2016/5936354 PMC4802015 27051450

[9] Liao MJ, Ye MN, Zhou RJ, Sheng JY, Chen HF. Yiqi formula enhances the antitumor effects of erlotinib for treatment of triple-negative breast cancer xenografts. Evidence-based complementary and alternative medicine : eCAM 2014. 2014:628712. 10.1155/2014/628712 PMC4217362 25389442

[10] Ma Y, Liu C, Qu D, Chen Y, Huang M, Liu Y (2017). Antibacterial evaluation of sliver nanoparticles synthesized by polysaccharides from Astragalus membranaceus roots. Biomed Pharmacother.

[11] Zhu J, Zhang H, Zhu Z, Zhang Q, Ma X, Cui Z, Yao T (2015). Effects and mechanism of flavonoids from Astragalus complanatus on breast cancer growth. Naunyn Schmiedeberg's Arch Pharmacol.

[12] Wang Y, Auyeung KK, Zhang X, Ko JK (2014). Astragalus saponins modulates colon cancer development by regulating calpain-mediated glucose-regulated protein expression. BMC Complement Altern Med.

[13] Fu J, Wang Z, Huang L, Zheng S, Wang D, Chen S, Zhang H, Yang S (2014). Review of the botanical characteristics, phytochemistry, and pharmacology of Astragalus membranaceus (Huangqi). Phytother Res.

[14] Denzler K, Moore J, Harrington H, Morrill K, Huynh T, Jacobs B, Waters R, Langland J. Characterization of the physiological response following in vivo administration of Astragalus membranaceus. Evidence-based complementary and alternative medicine : eCAM 2016. 2016:6861078. 10.1155/2016/6861078 PMC4844899 27190535

[15] Lian Y, Xie L, Chen M, Chen L. Effects of an astragalus polysaccharide and rhein combination on apoptosis in rats with chronic renal failure. Evidence-based complementary and alternative medicine : eCAM 2014. 2014:271862. 10.1155/2014/271862 PMC3966320 24711851

[16] Wang Z, Dong L, Zhen Y, Wang Y, Qi D, Xu A, Meng X, Li W (2016). Astragalus extract inhibits proliferation but enhances apoptosis in gastric cancer. BMC Complement Altern Med.

[17] Cheng XD, Gu JF, Yuan JR, Feng L, Jia XB (2015). Suppression of A549 cell proliferation and metastasis by calycosin via inhibition of the PKCalpha/ERK1/2 pathway: an in vitro investigation. Mol Med Rep.

[18] Huang WH, Liao WR, Sun RX (2016). Astragalus polysaccharide induces the apoptosis of human hepatocellular carcinoma cells by decreasing the expression of Notch1. Int J Mol Med.

[19] Tseng A, Yang CH, Chen CH, Chen CH, Hsu SL, Lee MH, Lee HC, Su LJ (2016). An in vivo molecular response analysis of colorectal cancer treated with Astragalus membranaceus extract. Oncol Rep.

[20] Wang SF, Wang Q, Jiao LJ, Huang YL, Garfield D, Zhang J, Xu L (2016). Astragalus-containing traditional Chinese medicine, with and without prescription based on syndrome differentiation, combined with chemotherapy for advanced non-small-cell lung cancer: a systemic review and meta-analysis. Current oncology (Toronto Ont).

[21] Lai X, Xia W, Wei J, Ding X (2017). Therapeutic effect of Astragalus polysaccharides on hepatocellular carcinoma H22-bearing mice. Dose-response: a publication of International Hormesis Society.

[22] Zhou Z, Meng M, Ni H (2017). Chemosensitizing effect of Astragalus polysaccharides on nasopharyngeal carcinoma cells by inducing apoptosis and modulating expression of Bax/Bcl-2 ratio and caspases. Medical science monitor: international medical journal of experimental and clinical research.

[23] Zhou R, Xu L, Ye M, Liao M, Du H, Chen H (2014). Formononetin inhibits migration and invasion of MDA-MB-231 and 4T1 breast cancer cells by suppressing MMP-2 and MMP-9 through PI3K/AKT signaling pathways. Horm Metab Res.

[24] Xu L, Luo J, Jin R, Yue Z, Sun P, Yang Z, Yang X, Wan W, Zhang J, Li S (2016). Bortezomib inhibits Giant cell tumor of bone through induction of cell apoptosis and inhibition of osteoclast recruitment, Giant cell formation, and bone resorption. Mol Cancer Ther.

[25] Zhang D, Zhuang Y, Pan J, Wang H, Li H, Yu Y, Wang D (2012). Investigation of effects and mechanisms of total flavonoids of Astragalus and calycosin on human erythroleukemia cells. Oxidative Med Cell Longev.

[26] Kumar S, Pandey AK (2013). Chemistry and biological activities of flavonoids: an overview. ScientificWorldJournal.

[27] Jin YM, Xu TM, Zhao YH, Wang YC, Cui MH (2014). In vitro and in vivo anti-cancer activity of formononetin on human cervical cancer cell line HeLa. Tumour Biol.

[28] Yang Y, Zhao Y, Ai X, Cheng B, Lu S (2014). Formononetin suppresses the proliferation of human non-small cell lung cancer through induction of cell cycle arrest and apoptosis. Int J Clin Exp Pathol.

[29] Azwanida NN, Review A (2015). On the extraction methods use in medicinal plants, principle, strength and limitation. Med Aromat Plants.

[30] Biesaga M (2011). Influence of extraction methods on stability of flavonoids. J Chromatogr A.

[31] Auyeung KK, Mok NL, Wong CM, Cho CH, Ko JK (2010). Astragalus saponins modulate mTOR and ERK signaling to promote apoptosis through the extrinsic pathway in HT-29 colon cancer cells. Int J Mol Med.

[32] Li LK, Kuang WJ, Huang YF, Xie HH, Chen G, Zhou QC, Wang BR, Wan LH (2012). Anti-tumor effects of Astragalus on hepatocellular carcinoma in vivo. Indian journal of pharmacology.

[33] He CS, Liu YC, Xu ZP, Dai PC, Chen XW, Jin DH (2016). Astragaloside IV enhances cisplatin Chemosensitivity in non-small cell lung cancer cells through inhibition of B7-H3. Cellular physiology and biochemistry: international journal of experimental cellular physiology, biochemistry, and Pharmacology.

[34] Wang DQ, Li Y, Tian YP, Wang CB (2005). Inhibition effects of total flavonids of Astragalus on BEL-7402 cell in vitro. Academic Journal of PLA Postgraduate Medical School.

[35] Fulda S, Debatin KM (2006). Extrinsic versus intrinsic apoptosis pathways in anticancer chemotherapy. Oncogene.

[36] Arcaro A, Guerreiro AS (2007). The phosphoinositide 3-kinase pathway in human cancer: genetic alterations and therapeutic implications. Curr Genomics.

[37] Ghayad SE, Cohen PA (2010). Inhibitors of the PI3K/Akt/mTOR pathway: new hope for breast cancer patients. Recent Pat Anticancer Drug Discov.

[38] Porta C, Paglino C, Mosca A (2014). Targeting PI3K/Akt/mTOR signaling in cancer. Front Oncol.

[39] Gonzalez-Angulo AM, Blumenschein GR (2013). Defining biomarkers to predict sensitivity to PI3K/Akt/mTOR pathway inhibitors in breast cancer. Cancer Treat Rev.

