# Supplementary material for: Extract from Astragalus membranaceus inhibit breast cancer cells proliferation via PI3K/AKT/mTOR signaling pathway
Source: BMC Complement Altern Med. 2018 Mar 9;18:83. doi: 10.1186/s12906-018-2148-2 (PMC5845298; doi:10.1186/s12906-018-2148-2)
Supplement: Supplementary file 1 — Figure S1. Cytotoxic effects of AM extract on three breast cancer cell lines for 24 h. After treated with different concentration of AM extract for 24 h, the cell prolieration was evaluated with MTT assay. Values are the mean ± S.D. of triplicate determinations of three independent experiments. **p < 0.01, ***p < 0.001, compared with blank control group (CTRL); #p < 0.05, ##p < 0.01, ###p < 0.001, compared with DMSO group. (PPTX 92 kb) [file 12906_2018_2148_MOESM1_ESM.pptx]

## Slide 1
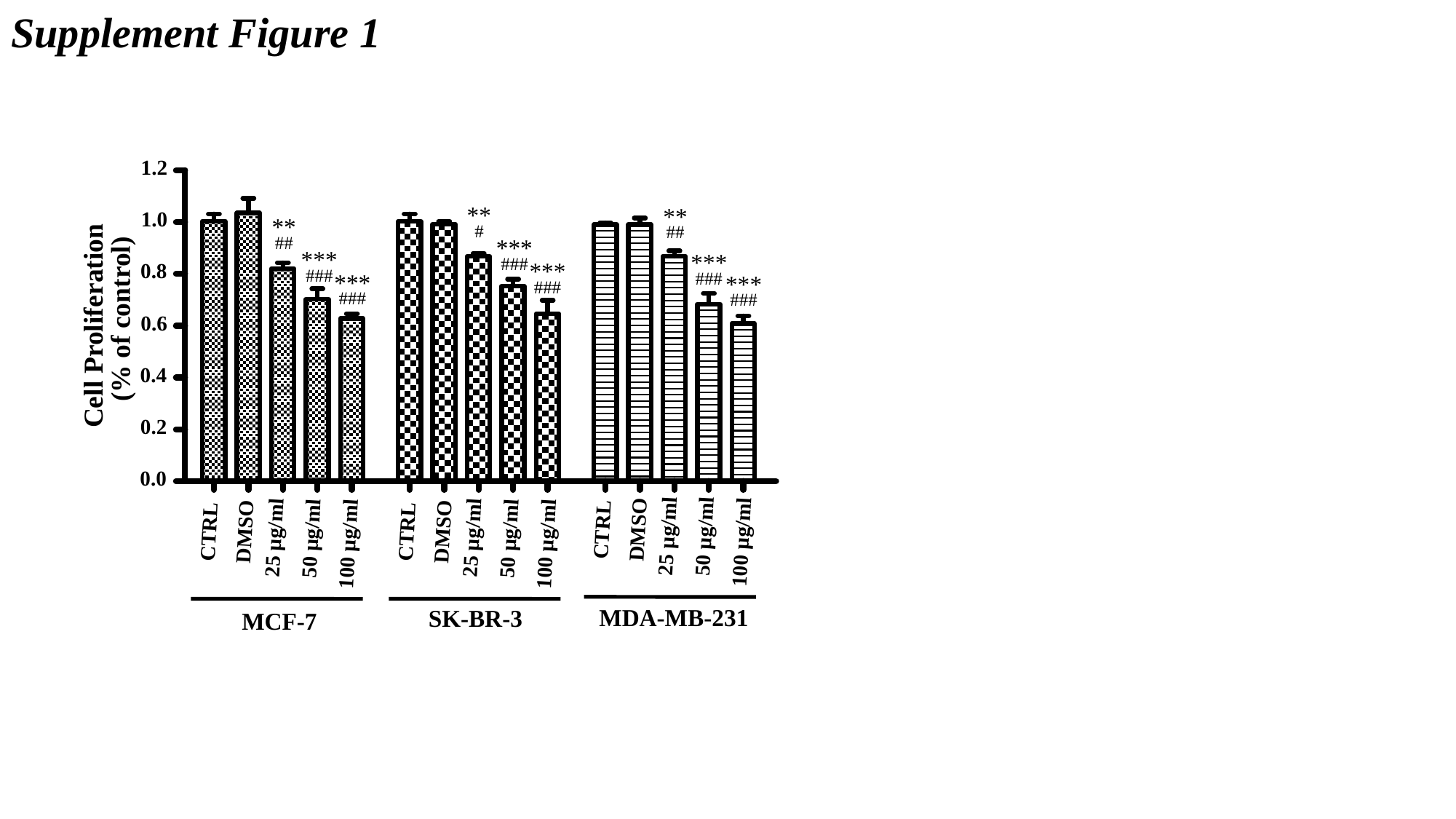

Supplement Figure 1
1.2
1.0
**
#
**
##
**
##
***
###
0.8
***
###
***
###
***
###
***
###
***
###
Cell Proliferation
 (% of control)
0.6
0.4
0.2
0.0
DMSO
CTRL
DMSO
DMSO
CTRL
CTRL
25 μg/ml
50 μg/ml
25 μg/ml
25 μg/ml
50 μg/ml
50 μg/ml
100 μg/ml
100 μg/ml
100 μg/ml
MDA-MB-231
SK-BR-3
MCF-7
