# Supplementary figures and images for: Extract from Astragalus membranaceus inhibit breast cancer cells proliferation via PI3K/AKT/mTOR signaling pathway
Source: BMC Complement Altern Med. 2018 Mar 9;18:83. doi: 10.1186/s12906-018-2148-2 (PMC5845298; doi:10.1186/s12906-018-2148-2)

## Slide 1
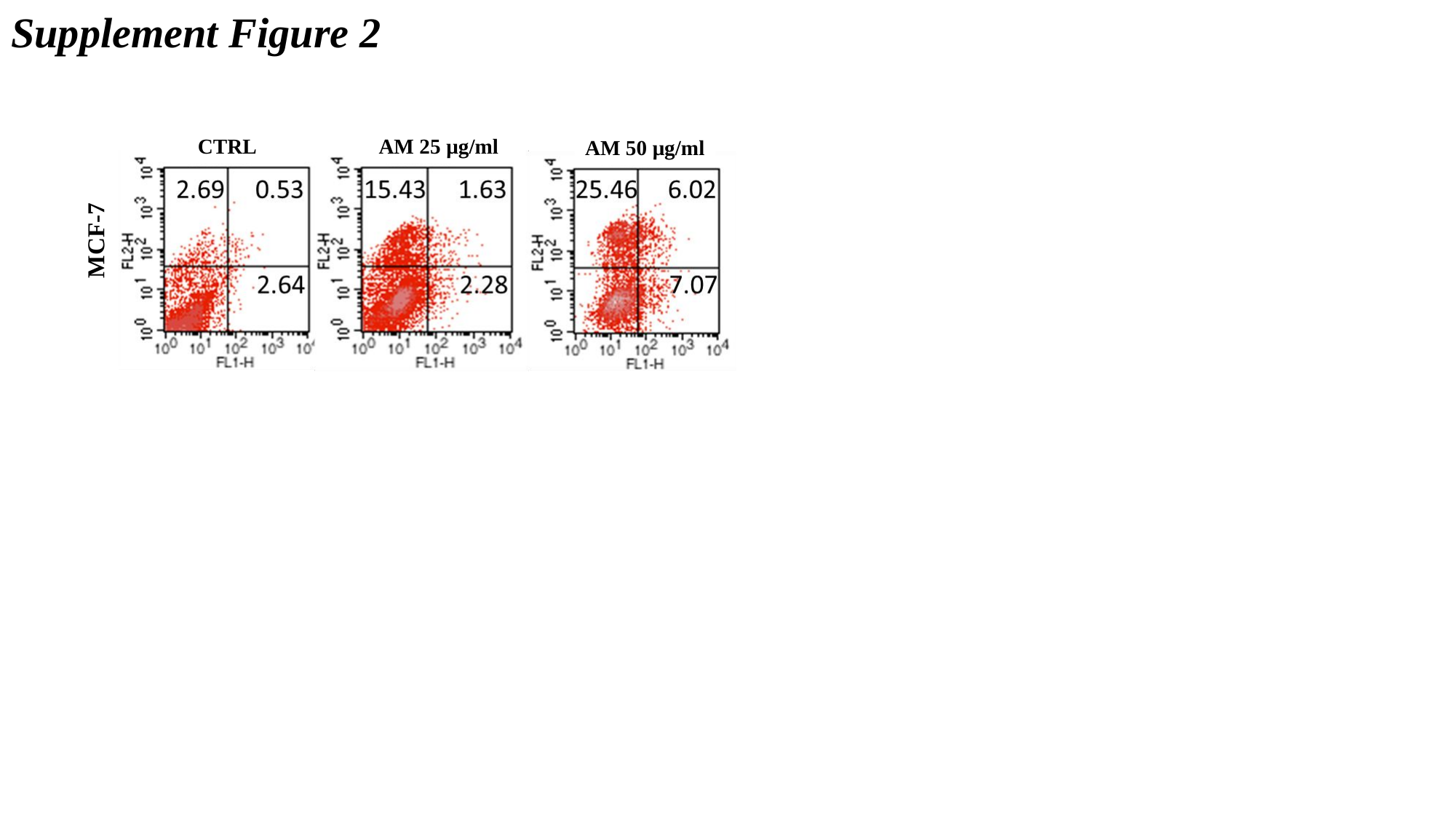

Supplement Figure 2
CTRL
AM 25 μg/ml
AM 50 μg/ml
MCF-7

Supplement: Supplementary file 2 — Figure S2. Analysis of cell apoptosis induced by AM extract in MCF-7 for 48 h. MFC-7 breast cancer cells were treated with 25 μg/ml and 50 μg/ml of AM extract for 48 h, and cell apoptosis was assessed by flow cytometry with FITC-Annexin V/PI Staining. (PPTX 262 kb) [file 12906_2018_2148_MOESM2_ESM.pptx]
